# Vicinal glutamates are better phosphomimetics: Phosphorylation is required for allosteric activation of guanylyl cyclase-A

**DOI:** 10.3389/fnmol.2022.1012784

**Published:** 2022-11-04

**Authors:** Neil M. Otto, Lincoln R. Potter

**Affiliations:** Department of Biochemistry, Molecular Biology and Biophysics at the University of Minnesota, Minneapolis, MN, United States

**Keywords:** phosphorylation, cyclic GMP, guanylyl cyclase, hypertension, heart failure, natriuretic peptide

## Abstract

Multisite phosphorylation of guanylyl cyclase (GC)-A, also known as NPR-A or NPR1, is required for receptor activation by natriuretic peptides (NPs) because alanine substitutions for the first four GC-A phosphorylation sites produce an enzyme that cannot be stimulated by NPs. In contrast, single Glu substitutions for the first six chemically identified GC-A phosphorylation sites to mimic the negative charge of phosphate produced an enzyme that is activated by NPs but had an elevated Michaelis constant (Km), resulting in low activity. Here, we show that vicinal (double adjacent) Glu substitutions for the same sites to mimic the two negative charges of phosphate produced a near wild type (WT) enzyme with a low Km. Unlike the enzyme with single glutamate substitutions, the vicinally substituted enzyme did not require the functionally identified Ser-473-Glu substitution to achieve WT-like activity. Importantly, the negative charge associated with either phosphorylation or glutamate substitutions was required for allosteric activation of GC-A by ATP. We conclude that vicinal Glu substitutions are better phosphomimetics than single Glu substitutions and that phosphorylation is required for allosteric activation of GC-A in the absence and presence of NP. Finally, we suggest that the putative functionally identified phosphorylation sites, Ser-473 in GC-A and Ser-489 in GC-B, are not phosphorylation sites at all.

## Introduction

**Figure 1:**
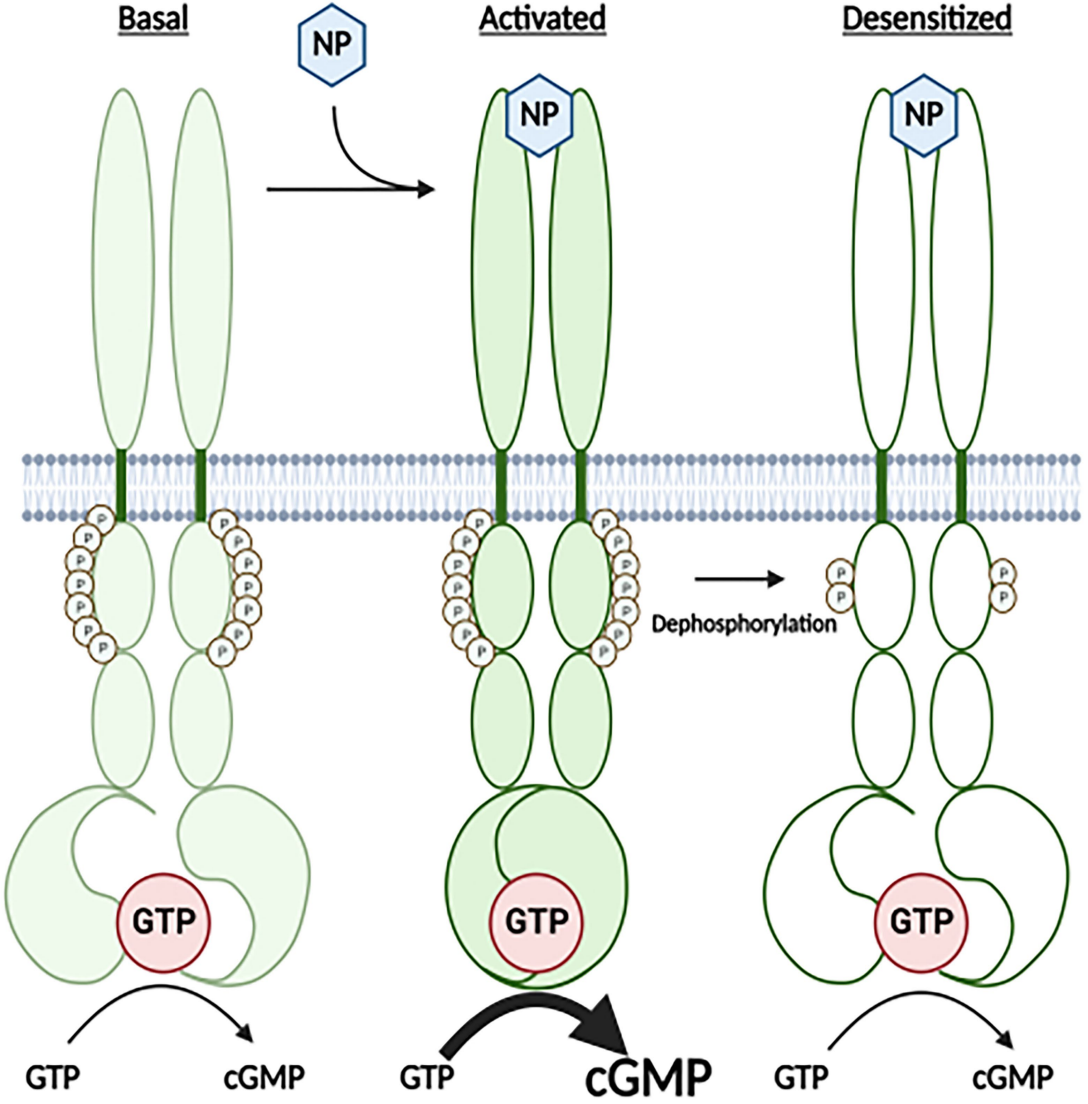
A Cartoon Model for how the highly phosphorylated version GC-A is activated by natriuretic peptide and how prolonged NP binding results in the dephosphorylation and inactivation of the receptor. Under basal conditions, GC-A is highly phosphorylated and has low GC activity. Once bound by natriuretic peptide (NP), affinity of the enzyme for GTP dramatically increases, which results in increased cGMP synthesis. With time the NP-bound receptor is dephosphorylated, which results in decreased affinity of the GC domain for GTP and reduced cGMP formation.

Atrial natriuretic peptide (NP) and B-type NP reduce blood pressure, blood volume, heart size and metabolism by activating guanylyl cyclase (GC)-A (Potter et al., 2006; Bordicchia et al., 2012; Kuhn, 2016). C-type natriuretic peptide (CNP) activation of the homologous enzyme, GC-B, regulates female reproduction, long bone growth and density, neuron bifurcation and other processes (Potter, 2011a,b; Kuhn, 2016; Jaffe and Egbert, 2017). Both GC-A and GC-B are homo-dimeric, single membrane-spanning enzymes that contain a glycosylated, extracellular ligand binding domain, a single membrane-spanning region and multi-domain intracellular region consisting of a serine and threonine phosphorylated kinase homology domain, dimerization domain and C-terminal catalytic domain (Figure 1; Potter et al., 2009; Potter, 2011a; Kuhn, 2016). GC-A and GC-B are synthesized by the ribosome, glycosylated in the E. R. and glycosylated again in the Golgi apparatus (Dickey et al., 2016). At this point, the KHD can be phosphorylated, which is required for activation of the GC domain by natriuretic peptide binding (Koller et al., 1993; Dickey et al., 2016). As a result of the extensive post-translational processing, GC-A is often found in as many as three species when purified by immunoprecipitation followed by SDS-PAGE. Importantly, it is only the highest MW, fully glycosylated species that is phosphorylated in these gels (Koller et al., 1993).

The kinase homology domain of GC-A, as well as the same domains in GC-B, GC-C, and GC-E, bind to and are allosterically regulated by ATP (Jewett et al., 1993; Parkinson et al., 1994; Foster and Garbers, 1998; Jaleel et al., 2006; Bereta et al., 2010; Robinson and Potter, 2012; Edmund et al., 2019), which is consistent with known functions of other pseudokinase domain containing enzymes (Murphy et al., 2014; Eyers and Murphy, 2016). Both GC-A and GC-B are maximally phosphorylated and maximally responsive to NPs in serum-starved cells (Potter and Garbers, 1992; Koller et al., 1993; Potter, 1998; Joubert et al., 2001), but prolonged NP exposure or brief exposure to chemical activators of protein kinase C or hormones or growth factors that antagonize the actions of NPs cause the dephosphorylation and inactivation GC-A and GC-B in cell culture (Figure 1; Chrisman and Garbers, 1999; Muller et al., 2006; Egbert et al., 2014; Robinson et al., 2017).

Six phosphorylation sites were initially identified by tryptic phosphopeptide mapping of ^32^PO_4_ labeled GC-A protein isolated from transfected 293 cells (Potter and Hunter, 1998b). Later, two groups identified the same six sites and one new site, Ser-487, by mass spectrometry (Schroter et al., 2010; Yoder et al., 2010). Mutation of four or more phosphorylation sites to Ala to mimic a dephosphorylated residue resulted in an enzyme that bound NP but could not transmit the NP binding signal to the catalytic domain (Potter and Hunter, 1998b). Conversely, single Glu substitutions for the same six sites resulted in an enzyme that was activated by NPs but to a much lower level than that of the phosphorylated WT enzyme (Potter and Hunter, 1999). Later, a functional screen identified a conserved juxta-membrane serine, Ser-473 in GC-A (Yoder et al., 2012), that when mutated to glutamate to make GC-A-8E resulted in a dephosphorylated enzyme with enzymatic characteristics like the phosphorylated WT enzyme (Otto et al., 2017). However, no physical evidence for the phosphorylation of Ser-473 in GC-A, or the corresponding residue, Ser-489, in GC-B (Yoder et al., 2012), has been found despite extensive attempts, which cast doubt on whether Ser-473 or Ser-489 are actually phosphorylated in a biological setting.

The first and most common phosphomimetic amino acid substitutions were single glutamates or single aspartates, both of which have a charge of −1 (Thorsness and Koshland, 1987). However, phosphorylated amino acids can have a charge between −1 and −2 depending on the surrounding pH, since phosphates have 3 pKas of 2.2, 7.2, and 12.4 (Hunter, 2012). The cytosolic pH of HEK 293T cells that were used for most of the phosphorylation studies on GC-A and GC-B is 7.4 (Matsuyama et al., 2000), so a charge close to −2 is expected for phosphorylated amino acids. Here, we replicated the −2 charge of phosphate by using vicinal Glu substitutions. We show that vicinal Glu substitutions for the seven definitive, chemically, identified sites produces a WT like enzyme that is no longer regulated by the Ser-473-Glu substitution. Finally, maximal activation of GC-A requires ANP binding to the extracellular domain and ATP binding to an allosteric site or sites in the intracellular domain (Joubert et al., 2005; Antos and Potter, 2007; Burczynska et al., 2007; Robinson and Potter, 2012; Edmund et al., 2019). Phosphorylation is required for ANP-dependent activation of GC-A, but previous studies failed to detect effects of phosphorylation on basal activity (Potter and Garbers, 1992; Potter and Hunter, 1998b). In contrast, phosphorylation is required for allosteric regulation of the homologous sea urchin GC receptor (Ramarao and Garbers, 1988). Here, we show that allosteric activation of GC-A under basal and NP-stimulated conditions requires phosphorylation of the chemically identified phosphorylation sites. Finally, our data indicate that the “functionally identified” phosphorylation sites in GC-A (S473) and GC-B (S489), are unlikely to phosphorylated under biologic conditions.

## Experimental

### Reagents

^125^I-cGMP radioimmunoassay kits were from Perkin Elmer (Waltham, MA) and unlabeled NPs were from Sigma (St. Louis, MO). Protease inhibitor tablets were from Roche, Inc. (Baltimore, MD).

### Mutagenesis

Ala or Glu substitutions for single or multiple phosphorylation sites were generated on the CMV3-GC-A plasmid as previously described (Potter and Hunter, 1998b).

### Transient transfection

Human embryonic kidney 293T cells were transiently transfected with 5 μg of pCMV3-GC-A plasmids containing single or multiple phosphorylation site mutations by the HEPES-calcium-phosphate precipitation method as previously described (Yoder et al., 2012).

### Plasma membrane preparation

Cells cultured on 10 cm plates were placed in serum-free media for 4 h before membranes were prepared. Membranes were harvested at 4°C by washing the plates twice with phosphate buffered saline, scraping the cells off the plates in 0.6 ml phosphatase inhibitor buffer (PIB; Antos et al., 2005). Cells were lysed by sonication and the lysates were centrifuged at 20,000*g* for 15 min at 4°C. The supernatant was aspirated and the membrane pellet was resuspended with 0.5 ml PIB and centrifuged again at 20,000*g* for 15 min at 4°C. The supernatant was aspirated, and the pellet was resuspended in PIB to yield a protein concentration between 1 and 3 mg/ml. Crude membranes were assayed for GC activity without freezing.

### Guanylyl cyclase assays

Crude membranes were assayed for GC activity at 37°C in a buffer containing 0.5 mM isomethylxanthine to inhibit phosphodiesterases, 5 mM MgCl_2_, and a nucleotide regenerating system as reported (Robinson and Potter, 2012). 0.02 ml of crude membranes were added per assay. A solution of 100 mM GTP and 100 mM MgCl_2_ was diluted to the appropriate substrate concentrations used for each assay. For assays including NPs, assays were conducted for 5 min. For assays without NPs, assays were conducted for 10 min to allow enough cGMP generation to be detected by the radioimmunoassay. Because enzymatic activity was not completely linear with time, the kinetic parameters are considered “apparent.” Since GC-A activity in these experiments was dependent on transfection efficiency as well as proper post-translational modification of the receptor by processes that can be overwhelmed by increased transfection efficiency, kinetic parameters can vary between individual experiments. Hence, comparisons between wild type and mutant enzymes were determined in the same assay that was repeated multiple times in order to achieve statistical significance.

### SDS-PAGE and gel staining

Eight percent resolving gels were fixed in a 30 ml solution of 50% methanol and 10% acetic acid for 30 min with gentle rocking. The solution was changed two times for a total of three washes in the fixing solution. The gels were then washed twice in 100 ml of water for 10 min. Ten milliliters of Pro-Q Diamond phosphoprotein gel stain was added, and the gels were incubated with gentle rocking for 1.5 h in the dark. The gels were then destained with 80 ml of a solution of 20% acetonitrile and 50 mM sodium acetate (pH 4.0) for 15 min. This wash was repeated two times for a total of three washes. The gels were then rinsed in water, scanned with a 532 nm laser, and imaged with FUJI FLA 5000 software as previously described (Otto et al., 2017). After imaging, the same gels were stained with 50% Methanol, 7% Acetic Acid, and 0.1% Coomassie Brilliant Blue for 10 min and destained in a solution of 50% methanol and 7% acetic acid.

### Statistical analysis

Substrate-velocity curves were analyzed by nonlinear regression with an allosteric sigmoidal model in Prism 7 to determine the Vmax, Km, and Hill coefficients. A Michaelis–Menten model was used to determine Vmax and Km in the presence of ATP. “Km is represented by K half in some figures.” For each pair of enzymes and each model parameter, we compared the full model fit against a null model in which that parameter was set to be equal. Because of this, we used the extra sum of squares F test to generate *p*-values. Unpaired *t*-tests were used to analyze single substrate GC assays. A significant difference in all tests was where *p* < 0.05. Horizontal bars above and below individual data points represent the standard error of the mean. When no vertical bars are shown, they are within the symbol itself.

## Results

### Vicinal glutamates are better phosphomimetics

We created GC-A-8E with 8 individual single Glu substitutions that has similar ligand activation (EC_50_) and enzymatic (Vmax and Km) characteristics to phosphorylated WT-GC-A (Otto et al., 2017). Importantly, we generated mice with these same substitutions for each GC-A allele that have smaller hearts but normal blood pressure compared to wild type mice (Wagner et al., 2022). However, the GC-A-8E mutant requires the Ser-473-Glu substitution, and there is no physical evidence that Ser-473 is phosphorylated in any living system (Yoder et al., 2012). One reason that the Ser-473-Glu substitution may be required is that the total negative charge for GC-A-8E is deficient since Glu with a charge of −1 was substituted for phosphoserines or phosphothreonines that have a charge closer to −2 in cells. If this hypothesis is true, then vicinal Glu substitutions that result in −2 charges for the chemically identified sites in GC-A should produce a WT-like enzyme without the need for the Glu substitution at Ser-473.

**Figure 2:**
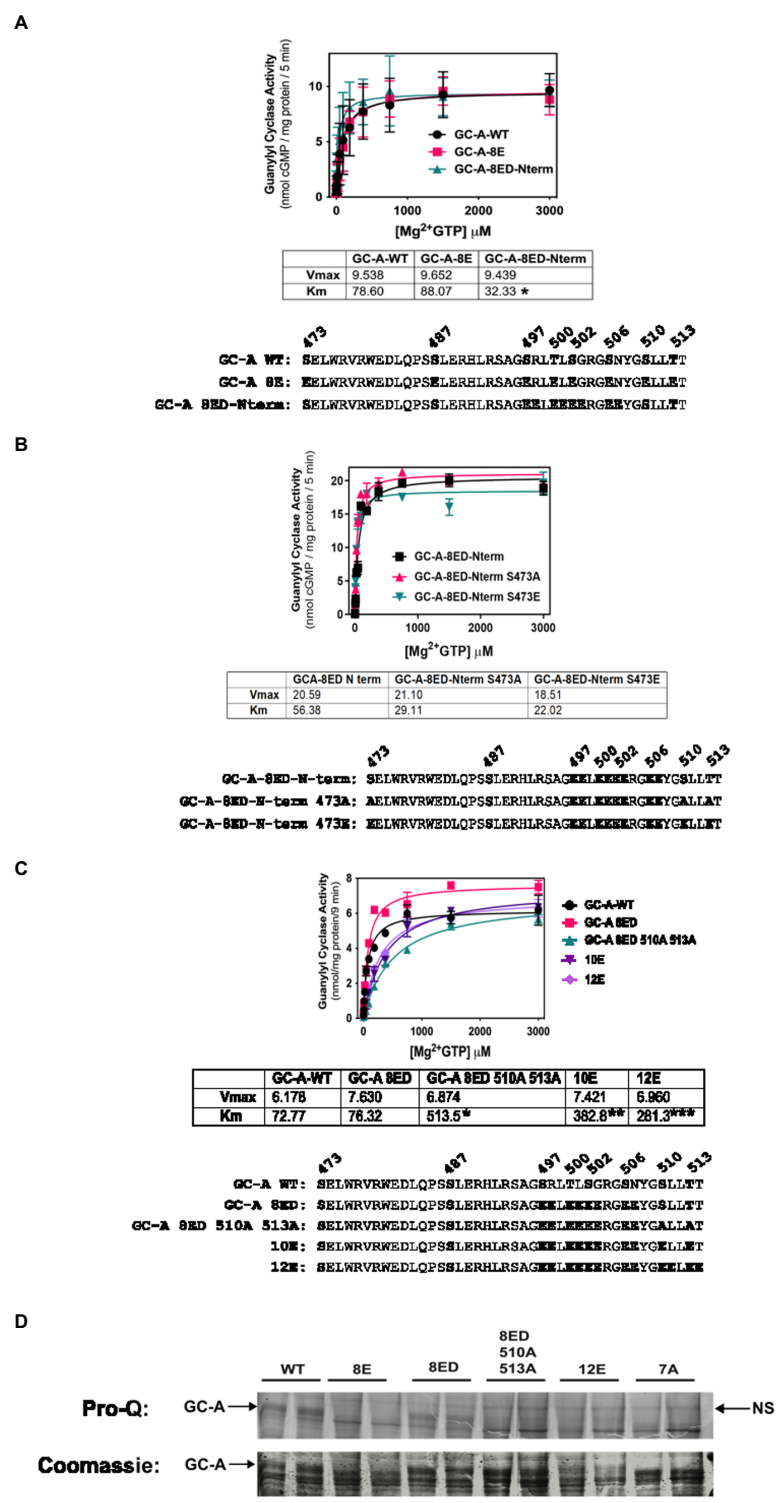
Vicinal Glu substitutions at Ser497, Thr-500, Ser-502 and Ser-506 produce a WT-like version of GC-A. 293T cells were transiently transfected with the indicated version of GC-A and membranes from these cells were assayed for GC activity for 5 min in the presence of 1 uM ANP, 1 mM ATP, and increasing concentrations of GTP. The amino acid sequences of the individual constructs used in each experiment are shown below each panel. **(A)** Comparison between GC-A-WT, GC-A-8E, and GC-A-8ED-N term where *n* = 4 from two experiments. * indicates that the Km of GC-A-8ED-Nterm is significantly lower than the Kms from the other two constructs. **(B)** Comparison between GC-A-8ED-Nterm, GC-A-8ED-Nterm-473E, GC-A-8ED-Nterm-473A, where *n* = 3 from three transfections. **(C)** Comparison between, GC-A-WT, GC-A-8ED-Nterm, GC-A-8ED-Nterm-510A and 513A, GC-A-10E, and GC-A-12E where *n* = 3 from three transfections. * indicates that the Km of GC-A-Nterm-510A and 513A is significantly different from those of both GC-A-WT and GC-A-8ED-Nterm. ** indicates that the Km of GC-A-10E is significantly different from the Km of GC-A-Nterm-510A and 513A. *** indicates that the Km of 12E is significantly different from GC-A-10E. All differences are at *p* < 0.05 significance. **(D)** The indicated receptor constructs were transfected into 293T cells, then immunoprecipitated, fractionated by SDS-PAGE and stained with ProQ Diamond (top panel) to determine receptor phosphate content followed by Coomassie staining (bottom panel) to measure receptor protein levels. The arrows indicate the location of phosphorylated GC-A. NS next to the right-side arrow means non-specific band.

Initially, we made vicinal Glu substitutions at the first four N-terminal phosphorylation sites that were identified by tryptic phosphopeptide mapping (Potter and Hunter, 1998b). This mutant called GC-A-8E double amino-terminus, or GC-A-8ED-Nterm for short, contains Glu substitutions at Ser-497, Arg-498, Thr-500, Leu-501, Ser-502, Gly-503, Ser-506, and Asn-507. Substrate-velocity profiles for GC-A-8ED-Nterm, GC-A-8E and phosphorylated WT-GC-A revealed similar activities for all three enzymes, although the Km was significantly lower for GC-A-8ED-Nterm compared to the other two enzymes (Figure 2A).

We then examined if the Ser-473-Glu mutation was still required for maximum activation of GC-A-8ED-Nterm and found that neither Ala or Glu substitutions for Ser-473 significantly affected the activity of this vicinally-substituted enzyme (Figure 2B). These data suggest that if GC-A has sufficient negative charge at the chemically identified phosphorylation sites, then additional negative charge at Ser-473 is no longer required for normal ANP-dependent GC activity.

Since Ser-510 and Thr-513 could be phosphorylated in the GC-A-8ED-Nterm mutant, we blocked the ability of these two residues to be phosphorylated by converting them to alanine to produce GC-A-8ED-Nterm 510A 513A, which resulted in a 6.7-fold increase in the Km (Figure 2C). In contrast, substituting Glu for Ser-510 and Lys-511 in GC-A-8ED-Nterm to make GC-A-10E as well as at Thr-513 and Thr-514 to make GC-A-12E, progressively reduced the Km. These mutagenesis data suggest that Ser-510 and Thr-513 are phosphorylated in GC-A-8ED, and that the negative charge associated with phosphorylation at these residues reduces the Km of GC-A.

To directly determine if Ser-510 and Thr-513 are phosphorylated in GC-A-8ED-Nterm, Pro-Q Diamond phosphate staining of SDS gels was performed (Figure 2D) as previously described (Bryan et al., 2006). This technique accurately measures the phosphate content of GC-A as indicated by dark, slightly diffuse band, that migrates around 130 KDa on an 8% SDS gel. We found that WT-GC-A was more phosphorylated than any mutant as expected. The mutant GC-A-8ED-Nterm, called 8ED in the figure, was also phosphorylated, although to a lesser extent than WT-GC-A, which was expected because it retains two sites that are obviously phosphorylated. Importantly, Ala substitutions at Ser-510 and Thr-513 reduced the phosphate signal of GC-A-8ED-Nterm, which indicates that Ser-510 and Thr-513 are phosphorylated in the 8ED mutant. As expected, GC-A-8E, GC-A-12E (a construct where vicinal Glu substitutions were incorporated for the first six sites identified), and GC-A-7A (a dephosphorylated mutant containing Ala substitutions for all 7 known chemically identified sites) stained poorly for ProQ Diamond but strongly for Coomassie blue, which indicates that these receptors are expressed but are not phosphorylated as expected. Together, data from all four panels of Figure 2 indicate that only the chemically identified phosphorylation sites are required to produce a hormonally responsive form of GC-A and that Ser-473 is not phosphorylated in a biological setting.

### Glu substitutions at Ser-472 in GC-A or Ser-488 in GC-B increases GC activity

**Figure 3:**
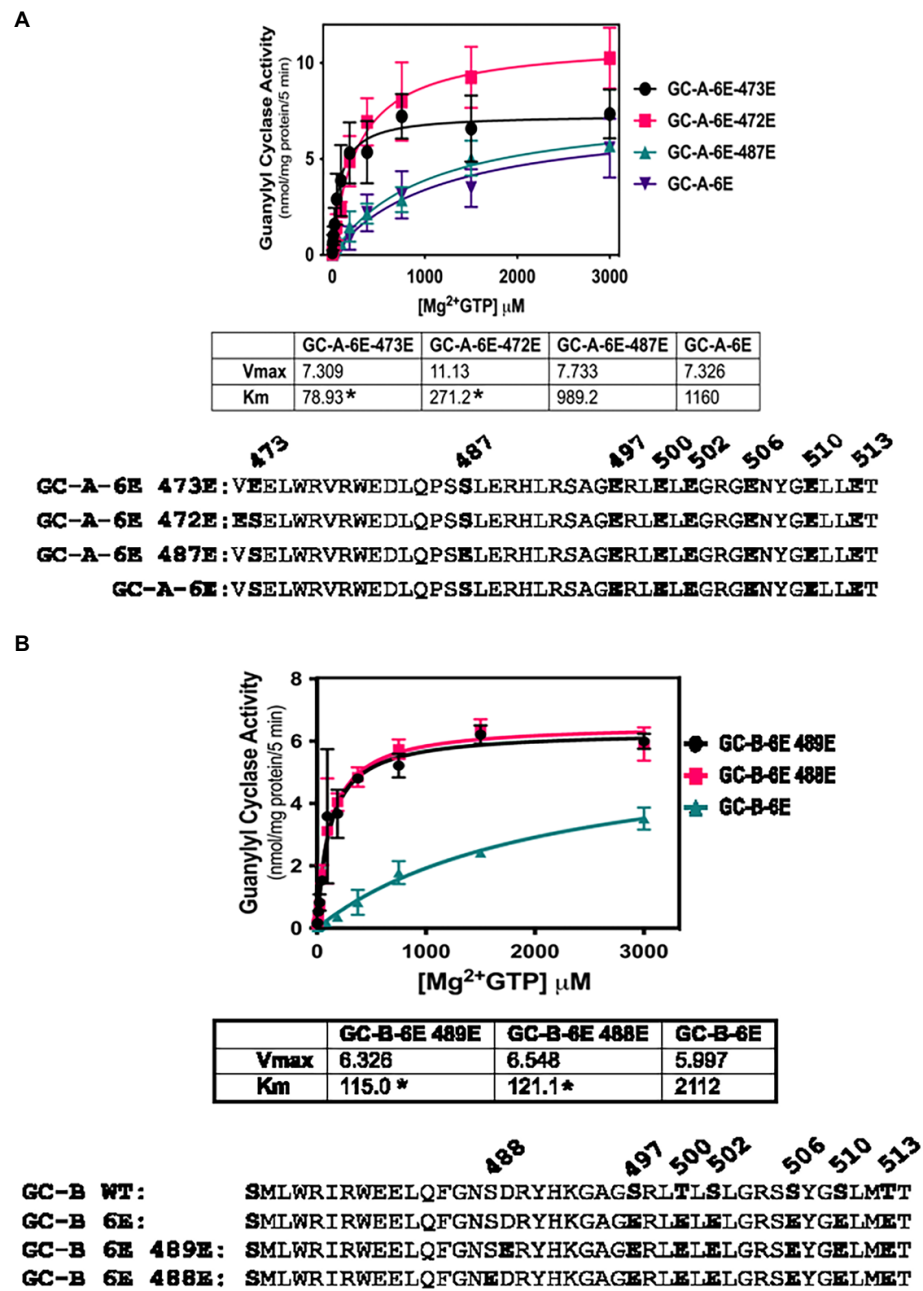
Activating Glu substitutions in juxta-membrane region of GC-A are not specific for Ser-473 in GC-A or Ser-489 in GC-B. 293T cells were transiently transfected with the indicated forms of GC-A or GC-B and membranes from these cells were assayed for GC activity for 5 min with 1 mM ATP, increasing concentrations of GTP and 1 uM ANP or CNP for GC-A and GC-B, respectively. **(A)** Comparison between GC-A-6E-473E, GC-A-6E-472E, and GC-A-6E, where *n* = 4. * indicates significantly different from GC-A-6E for each construct, respectively at *p* < 0.05. **(B)** Comparison between GC-B-6E-489E, GC-B-6E-488E, and GC-B-6E, where *n* = 4. * indicates significantly different from GC-B-6E for each construct, respectively at *p* < 0.05.

To further investigate the biological relevance of Ser-473 phosphorylation in GC-A as well as the corresponding residue, Ser-489 in GC-B, we tested whether negative charge at these exact residues is required for enzyme activation of 6E versions of GC-A and GC-B or whether introduction of negative charge at adjacent residues would also increase activity. We found that Glu substitutions at Val-472 dramatically reduced the Km of the GC-A-6E enzyme more than four-fold but the Glu substitution at Ser-473 reduced the Km to a greater extent for GC-A-6E, a construct containing single Glu substitutions for first six identified phosphorylation sites in GC-A (Figure 3A). Regarding GC-B, identical increases in activity were observed when Glu was substituted for either Ala-488 or Ser-489-Glu in GC-B-6E (Figure 3B). These data indicate that the introduction of negative charge in the general juxtamembrane region of the charge deficient, single Glu mutants of GC-A and GC-B, is sufficient to produce enzymes with WT like activity.

### Vicinal Glu substitutions at Ser-487 and Leu-488 are inhibitory

**Figure 4:**
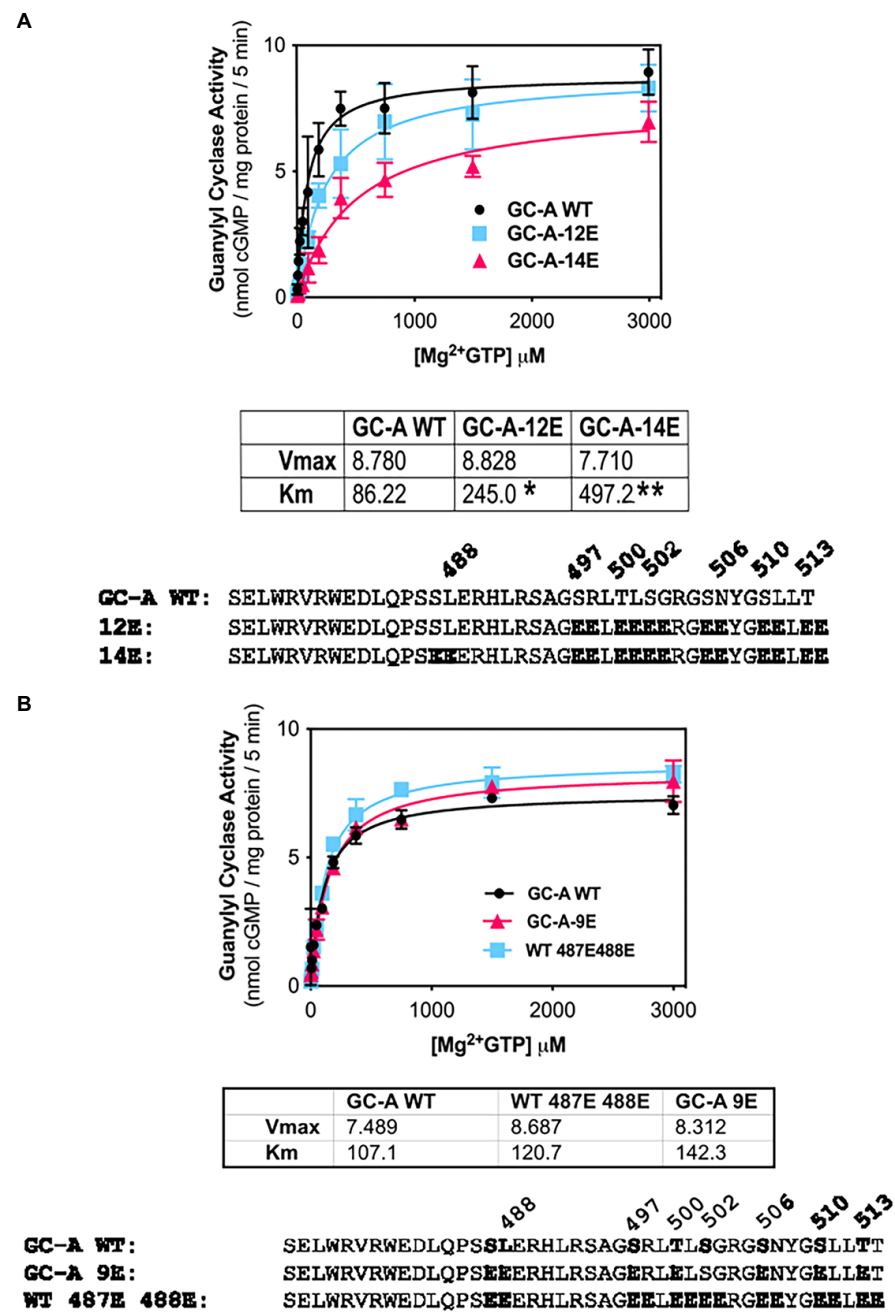
Vicinal Glu substitutions at Ser-487 and Leu-488 inhibit GC-A. **(A)** Comparison between, GC-A-WT, GC-A-12E, and GC-A-14E where *n* = 4. * indicates that GC-A-14E is significantly different from GC-A-WT. ** indicates that GC-A-14E is significantly different from GC-A-12E. All differences are at *p* < 0.05 significance. **(B)** Comparison between phosphorylated GC-A-WT, GC-A-9E, and GC-A-WT-487E-488E, where *n* = 4.

The initial report describing the Ser-487 phosphorylation site in GC-A, suggested that phosphorylation at this residue is inhibitory (Schroter et al., 2010). However, our efforts to create a dephosphorylated mutant of GC-A that has WT-like activity required the conversion of Ser-487 to Glu in GC-A-7E to make the single Glu-substitution mutant GC-A-8E (Otto et al., 2017), which is consistent with this site increasing activity. To determine whether the Ser-487 increases or decreased activity, we tested whether Glu substitutions at both Ser-487 and Leu-488 would increase or decrease the activity of various forms of GC-A. First, we examined whether converting Ser-487 and Leu-488 in GC-A-12E to make GC-A-14E would affect the Km of the enzyme and found that the Km of GC-A-14E was higher than that of both WT-GC-A and GC-A-12E, which is consistent with this site inhibiting activity (Figure 4A). We also examined what effect substituting a Glu for Leu-488 in GC-A-8E to make GC-A-9E has on activity. Note that this construct has vicinal Glu substitutions at Ser-487 and Leu-488 but single Glu substitutions for the other seven chemically identified sites for a total of nine glutamates. Additionally, we converted Ser-487 and Leu-488 to Glu in WT-GC-A. We found that adding the vicinal Glu substitutions at Ser-487 and Leu-488 tended to increase the Km of GC-A regardless of whether the negative charge was from natural phosphorylation sites or from single Glu substitutions, although none of these differences were statistically significant (Figure 4B). Hence, our best interpretation of the data from the vicinal substitutions, like the single substitution, suggests that the Ser-487 site inhibits GC activity as originally reported by Schroter et al. (2010).

### Phosphorylation is required for allosteric activation of GC-A

**Figure 5:**
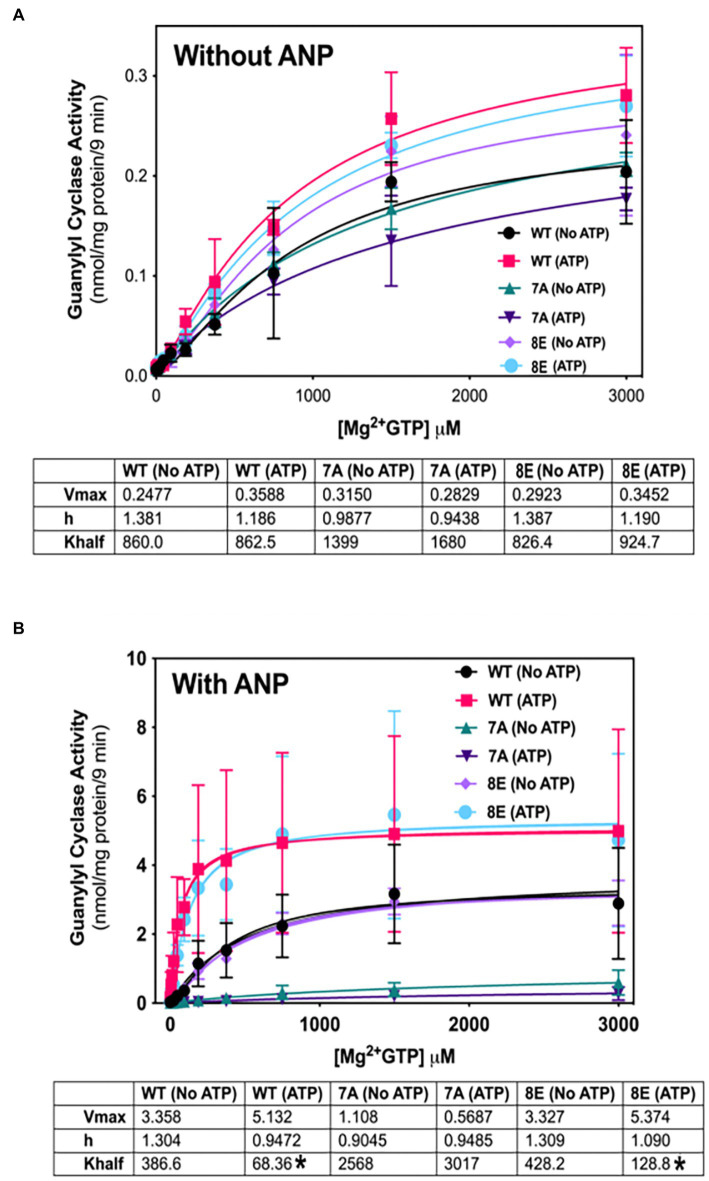
Phosphorylation is required for allosteric regulation of GC-A by NP and ATP. 293T cells were transiently transfected with the indicated GC-A constructs and membranes from these cells were assayed for GC activity for 9 min in the absence **(A)** or presence **(B)** of 1 υM ANP with or without 1 mM ATP with increasing concentrations of GTP. Comparison between WT-GC-A, GC-A-7A, and GC-A-8E in the presence or absence of 1 mM ATP where *n* = 3 from three transfections. * indicates significantly different from no ATP treatment for each receptor, respectively, at *p* < 0.05 significance.

NPs decrease the Km of GC-A and GC-B by a process involving ATP binding to a pseudosymmetric allosteric site in the catalytic domains (Antos and Potter, 2007; Robinson and Potter, 2012) and possibly the kinase homology domains (Joubert et al., 2005; Burczynska et al., 2007; Edmund et al., 2019) of these enzymes. To determine whether phosphorylation is required for allosteric activation of GC-A, we compared the ability of ATP to reduce the Km of phosphorylated WT-GC-A, the original phosphomimetic mutant GC-A-8E (S473E, S487E, S497E, T500E, S502E, S506E, S510E, T513E) as initially described (Otto et al., 2017), and the dephosphorylated mutant, GC-A-7A (S487A, S497A, T500A, S502A, S506A, S510A, and T513A) in the presence (Figure 5B) and absence (Figure 5A) of ANP. Please note that the Km is represented by “K half” in Figure 5. With ANP, ATP reduced the Hill slope and Km of phosphorylated WT-GC-A and GC-A-8E but had no effect on GC-A-7A, consistent with phosphorylation being required for allosteric regulation of GC-A. In the absence of ANP, ATP allosterically shifted product formation from positive cooperative to linear for both WT-GC-A and GC-8E as evidenced by reductions in the Hill slope of the enzymes (Figure 5A). However, the Hill slope for GC-A-7A was approximately 1 and it did not change regardless of whether ATP was included in the reaction. These data indicate that phosphorylation, solely due to its ability to increase local negative charge, is required for allosteric regulation of GC-A.

### Phosphorylation increases basal activity of GC-A in an ATP-dependent manner

**Figure 6:**
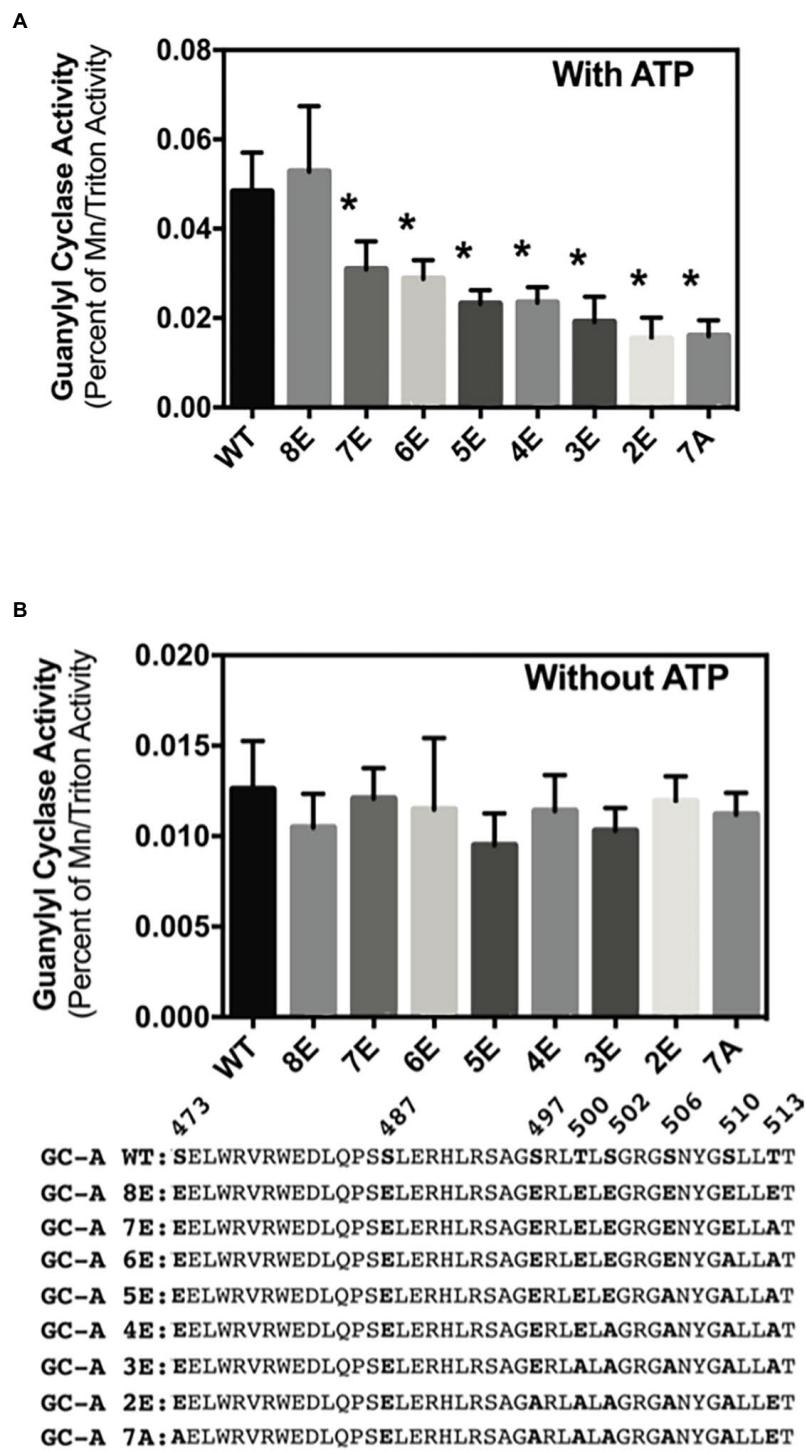
Phosphorylation increases ATP-dependent basal activity of GC-A. 293T cells were transiently transfected with the indicated GC-A constructs and membranes from these cells were assayed for GC activity for 5 min in the presence **(A)** or absence **(B)** of 1 mM ATP. All assays contained 0.1 mM GTP. Comparison between the indicated versions of GC-A where *n* = 10. * indicates significantly different from WT-GC-A at *p* < 0.05.

Phosphorylation of GC-A and GC-B is essential for NP stimulation of their catalytic domains, but initial experiments measuring cGMP production by the insensitive ^32^P-cGMP assay failed to detect effects of phosphorylation on basal GC activity (Potter, 1998; Potter and Hunter, 1998b). Experiments described here using a more sensitive radioimmunoassay to measure cGMP production indicated that phosphorylation increases basal GC activity for the first time. In the presence of ATP (cellular conditions), GC activity of phosphorylated WT-GC-A and the phosphomimetic mutant GC-A-8E did not differ (Figure 6A). However, incrementally decreasing the negative charge in GC-A-8E by converting single Glu to Ala, reduced the basal activity of the enzyme in a step-wise manner (Figure 6A). In contrast, without ATP the activities of all the GC-A mutants were the same regardless of the amount of negative charge from phosphate or glutamate (Figure 6B). These data indicate that increased negative charge resulting from phosphorylation increases basal activity of GC-A by an allosteric mechanism requiring ATP binding.

## Discussion

Single glutamate substitutions at all six chemically identified phosphorylation sites in GC-A yielded an enzyme called GC-A-6E that had a Km 11-fold higher than the phosphorylated WT enzyme. A possible explanation for the inability of single Glu substitutions to completely mimic the effects of phosphorylation sites is that they only have a negative charge of −1, whereas phosphoserines and phosphothreonines can have a negative charge as high as negative as −2 in cells. As originally employed by Strickfaden et al. (2007) who were studying the effect of phosphorylation of Ste5 on cell cycle regulation in yeast, we used vicinal Glu mutations to mimic the −2 charge of a phosphoserine or a phosphothreonine. Vicinal Glu mutations at all six originally identified sites produced an enzyme called GC-A-12E with a Km only 2.8 higher than the WT enzyme, which is a large improvement over the activity observed for GC-A-6E with single Glu substitutions. However, the Km of GC-A-12E was still slightly higher than the Km of the phosphorylated WT enzyme. Only GC-A-8ED-Nterm, with vicinal Glu mutations for the first four N terminal phosphorylation sites and phosphates on the two remaining residues produced a Km lower than that of the phosphorylated WT enzyme. In GC-A-8ED-Nterm, Ser-510 and Ser-513 are phosphorylated, yet vicinal Glu mutations at these same residues to produce GC-A-12E does not produce an enzyme with WT activity. Why GC-A-12E had slightly less activity than the WT enzyme may be explained by the negative charge on each Glu being in different orientations since they are on different carbons, which would not be the case with the natural phospho-amino acids since the negative charges from the hydroxyl groups are on the same phosphate. It is also not known if having double negative charges at each phosphorylation site is the optimal configuration for GC activity or whether the double glutamate substitutions produce other unforeseen conformations.

Another question addressed by these studies is why the Ser-473-Glu and Ser-489-Glu substitutions in GC-A and GC-B, respectively, are required to produced dephosphorylated enzymes with WT-like activity when single Glu substitutions are introduce for the known phosphorylation sites. This is especially important to understand given that there is no evidence that these residues are phosphorylated *in vivo.* The current study demonstrates that Ala and Glu substitutions at Ser-473 do not affect the activity of GC-A-8ED-Nterm. Furthermore, substituting an Glu for the Val at 472 in GC-A or Glu for Ala at 488 in GC-B, also reduced the Km of enzymes, which indicates that increased charge in the general vicinity of these conserved serines is only important when there is a charge deficiency at the chemically identified phosphorylation sites. Given that the only evidence supporting phosphorylation of Ser-473 in GC-A or Ser-489 in GC-B is based solely on activity changes, our best estimate is that neither Ser-473 nor Ser-489 are phosphorylated *in vivo*. Why Glu substitutions at Ser-473 and Ser-489 have disproportional effects on activity is not known, but they are in a region known to have a great effect on activity, since the GC-B Ala-488-Pro activating mutation that increases long bone growth is only one residue away (Miura et al., 2014).

To date, phosphorylation has only been shown to affect NP-stimulation of GC-A and GC-B, although recently we have been able to demonstrate the physiological importance of this process by showing that GC-B dephosphorylation is the mechanism by which luteinizing hormone stimulates resumption of meiosis in the oocyte as well as the mechanism by which fibroblast growth factor receptor-3 activation causes achondroplasia (Shuhaibar et al., 2016, 2017; Robinson et al., 2017; Wagner et al., 2021). Here, we show that phosphorylation increases basal activity for the first time. With the loss of each negative charge, there is a stepwise decrease in activity in the presence of ATP. However, in the absence ATP, each GC-A construct has similar activity regardless of charge, consistent with the increased activity resulting from allosteric activation by ATP. Our previous reports suggest that phosphorylation does not affect basal activity (Potter and Hunter, 1998a,b). However, these studies measured cGMP formation with a less sensitive ^32^P-GTP assay, which likely masked the differences in small amounts of cGMP produced in basal assays. Finally, the kinase or kinases that phosphorylate GC-A or GC-B have not been identified. However, LB-100, an inhibitor of PP2A and other similar phosphatases was recently shown to increased GC-B dependent long bone growth in mice (Shuhaibar et al., 2021), which suggest that phosphatase inhibitors may also increase GC-A activity *in vivo* as well.

In conclusion, we report that phosphorylation regulates basal activity and is required for allosteric regulation of GC-A by ATP, which is consistent with a report published more than 30 years earlier showing that phosphorylation is required for allosteric regulation of the homologous sea urchin GC receptor, the first member of the transmembrane GC family to be molecularly cloned (Ramarao and Garbers, 1988; Singh et al., 1988).

## Data availability statement

The original contributions presented in the study are included in the article/supplementary materials, further inquiries can be directed to the corresponding author.

## Author contributions

NO conducted experiments, analyzed and interpreted experimental data, and wrote the paper. NO and LP conceived and coordinated the study, analyzed and interpreted data, and wrote the paper. All authors contributed to the article and approved the submitted version.

## Funding

This work was supported by the National Institutes of Health grant R01GM098309 (to LP), The Fund for Science, and The Hormone Receptor Fund.

## Conflict of interest

The authors declare that the research was conducted in the absence of any commercial or financial relationships that could be construed as a potential conflict of interest.

## Publisher’s note

All claims expressed in this article are solely those of the authors and do not necessarily represent those of their affiliated organizations, or those of the publisher, the editors and the reviewers. Any product that may be evaluated in this article, or claim that may be made by its manufacturer, is not guaranteed or endorsed by the publisher.

## Abbreviations

cGMP: cyclic guanosine monophosphate
GC: guanylyl cyclase
Km: Michaelis constant
NP: natriuretic peptide
Vmax: maximal velocity
WT: wild type
